# Supplementary material for: Skin and Arterial Wall Deposits of 18F-NaF and Severity of Disease in Patients with Pseudoxanthoma Elasticum
Source: J Clin Med. 2020 May 8;9(5):1393. doi: 10.3390/jcm9051393 (PMC7290446; doi:10.3390/jcm9051393)
Supplement: Supplementary file 1 [file jcm-09-01393-s001.pdf]

Table S1: General data of patients.

**Body weight (Kg)**		**68 ± 11**
BMI (kg/mt^2)		26.8 ± 4,7
Systolic Blood pressure (mmHg)		129 ± 20
Diastolic blood pressure (mmHg)		77 ± 7
Ankle/brachial index	Right	0.90 ± 0.19
	Left	0.94 ± 0.15
	< 0.9	7 (39%)
Skin folds		
	Neck (right)	2 (2-3)
	Neck (left)	2 (2-3)
	Axilae (right)	2,5 (2-3)
	Axilae (left)	2,5 (2-3)
	Elbow (right)	2 (1-2)
	Elbow (left)	2 (1-2)
	Popliteal (right)	2 (0,75-2)
	Popliteal (left)	2 (0,75-2)
PHENODEX		
	Skin	3 (2-3)
	Eye	2 (2-3)
	Gastrointestinal	0 (0-0)
	Vascular	0 (0-0,15)
	Cardiac	0 (0-0)
Total PHENODEX		5,5 (4-6)
Hemoglobin (g/dL)		13.1 ± 1.1
VCM (fl)		90 ± 5
HCM (pg)		29 ± 2
RDW		13 ± 1
Platelets (mm3)		249 ± 49
Neutrophils (mm3)		4.30 ± 1.30
Glucose (mg/dL)		91 ± 10
Creatinine (mg/dL)		0.69 ± 0.13
ALT (UI/L)		31 ± 20
Cholesterol (mg/dL)		182 ± 51
HDL cholesterol (mg/dL)		67 ± 13
LDL cholesterol (mg/dL		96 ± 44
Triglycerides (mg/dL)		97 ± 60
TSH (µUI/mL)		1.81 ± 1.1
Calcium (mg/dL)		8.99 ± 0.43
Phosphate (mg/dL)		3.4 ± 0.43

Data are shown as number (%), mean ± SD or median (IQR).

Table S2 18F-NaF TBRmax and TBRmean in vessels and skin according to the severity of skin, eye and vascular stage according to the Phenodex score and the presence of nephrolithiasis.

	**Averaged Skin**		Averaged Vascular		Averaged Calcium Score
**Phenodex**	**TBRmax**	TBRmean	TBRmax	TBRmean	
Skin					
1	0.97 (0.97-0.97)	0.71 (0.71-0.71)	1.63 (1.63-1.63)	1.58 (1.58-1.58)	4.92 (4.92-4.92)
2	4.79 (2.47-8.57)	2.94 (1.65-5.64)	2.09 (1.98-2.82)	1.73 (1.56-2.34)	78 (0.51-255)
3	4.22 (3.22-8.00)	2.87 (2.22-5.67)	2.62 (2.28-2.80)	2.15 (1.86-2.34)	81 (0.84-339)
Eye					
1	0.97 (0.97-0.97)	0.71 (0.71-0.71)	1.63 (1.63-1.63)	1.58 (1.58-1.58)	4.92 (4.92-4.92)
2	4.79 (3.49-8.58)	2.94 (2.41-5.62)	2.50 (1.98-2.77)	2.04 (1.56-2.31)	40 (0.38-267)
3	3.63 (2.22-5.55)	2.38 (1.67-4.15)	2.63 (2.49-3.03)	2.25 (1.93-2.59)	255 (43-494)
Vascular [1]					
0	3.85 (2.20-8.27)	2.53 (1.67-5.31)	2.44 (1.98-2.78)	1.93 (1.68-2.33)	1.82 (0.51-78)
1	8.57 (3.52-13.6)	6.08 (2.64-9.53)	2.27 (1.78-2.77)	1.90 (1.49-2.32)	302 (302-302)
2	5.26 (2.95-6.71)	3.49 (1.94-4.92)	2.58 (2.21-2.84)	2.21 (1.71-2.39)	447 (279-1241)
Nephrolithiasis					
0	4.07 (2.40-6.53)	2.64 (1.63-4.21)	2.56 (1.88-2.87)	2.16 (1.57-2.39)	118 (1.60-290)
1	7.04 (2.87-11.3)	5.22 (2.05-7.66)	2.38 (2.01-2.62)	1.91 (1.73-2.11)	2.46 (0.12-407)

[1] $p < 0.01$ for Calcium Score column. Data shown as median (IQR).

Table S3. Skin TBRmax and TBRmean in the areas according to the clinical severity (range 0-3).

	**Right**		Left	
	**TBRmax**	TBRmean	TBRmax	TBRmean
Neck				
1	1.05 (1.05-1.05)	0.79 (0.79-0.79)	1.15 (1.15-1.15)	0.97 (0.97-0.97)
2	5.77 (3.18-12.4)	4.14 (2.29-9.31)	5.30 (3.27-12.09)	3.53 (2.70-8.54)
3	7.57 (4.27-17.87)	5.57 (3.00-9.48)	8.18 (3.40-16.40)	6.69 (2.22-9.08)
Axillae				
1	4.02 (3.21-4.82)	3.34 (3.28-3.40)	4.67 (3.52-5.82)	3.27 (2.52-4.01)
2	9.92 (5.21-17.20)	5.71 (2.96-9.48)	10.14 (3.48-17.7)	6.25 (2.15-10.81)
3	6.09 (3.31-7.86)	4.62 (2.27-5.58)	5.26 (2.55-7.48)	3.52 (1.82-5.41)
Elbow				
0	0.74 (0.74-0.74)	0.55 (0.55-0.55)	0.60 (0.60-0.60)	0.51 (0.51-0.51)
1	1.12 (0.83-1.47)	0.92 (0.65-1.01)	0.96 (0.40-1.06)	0.71 (0.33-0.86)
2	3.74 (2.18-5.56)	2.73 (1.60-3.87)	3.81 (1.82-6.02)	2.46 (1.93-3.21)
Poplit				
0	2.49 (1.21-7.68)	1.74 (0.87-5.63)	2.32 (1.48-8.81)	1.56 (1.13-6.14)
1	2.70 (1.23-4.89)	2.12 (0.69-3.46)	2.54 (1.26-5.74)	2.04 (0.73-3.51)
2	4.66 (2.90-5.43)	2.78 (1.70-3.91)	3.36 (2.81-4.60)	2.46 (1.93-3.21)

Data shown as median (IQR).

Table S4. TBRmax and TBRmean in vascular territories according to the presence of vascular calcification.

Territory	N	Calcium Score	TBR max	TBRmean
Right Carotid				
No	14	0	1.70 (1.35-2.05)	1.54 (1.20-1.79)
Yes	4	363 (163-1041)	2.22 (1.52-3.17)	1.95 (1.46-2.56)
Left Carotid				
No	13	0	1.81 (1.39-2.13)	1.59 (1.22-1.75)
Yes	5	281 (18-723)	2.34 (1.74-3.09)	1.96 (1.50-2.54)
Ascending				
No	14	0	1.48 (1.39-2.01)	1.19 (1.09-1.60)
Yes	4	45 (43-49)	1.66 (1.65-1.86)	1.30 (1.27-1.48)
Aortic Arch				
No	8	0	2.03 (1.59-2.65)	1.66 (1.26-2.19)
Yes	10	31 (10-514)	2.16 (1.82-2.29)	1.69 (1.42-1.81)
Desc. aorta				
No	12	0	2.62 (2.19-3.08)	1.98 (1.75-2.69)
Yes	6	711 (56-1827)	3.21 (2.22-5.31)	2.62 (1.74-4.60)
Abd. Aorta				
No	9	0	3.74 (2.50-4.77)	3.30 (2.55-3.75)
Yes	9	440 (191-4398)	4.00 (3.46-5.33)	3.33 (2.93-5.07)
Right Iliac [1]				
No	10	0	1.94 (1.21-2.10)	1.67 (1.04-1.91)
Yes	8	680 (231-2055)	2.77 (2.19-3.38)	2.00 (0.95-2.74)
Left Iliac				
No	10	0	1.63 (1.35-2.38)	1.51 (1.13-1.96)
Yes	8	309 (112-1860)	2.22 (1.86-3.37)	1.76 (1.39-2.82)
Right Femoral				
No	8	0	3.11 (2.48-3.41)	2.68 (2.14-2.94)
Yes	10	469 (206-1485)	2.63 (2.19-3.35)	1.95 (1.56-2.84)
Left Femoral				
No	9	0	2.89 (2.51-3.91)	2.45 (2.03-3.27)
Yes	9	594 (273-1686)	2.85 (2.17-3.73)	2.05 (1.82-2.79)
Right Popl [3, 4]				
No	6	0	1.27 (0.88-1.80)	1.0 (0.76-1.35)
Yes	12	318 (13-815)	1.82 (1.37-2.25)	1.51 (1.21-1.86)
Left Popl [1,2]				
No	7	0	1.10 (0.88-1.61)	0.95 (0.78-1.31)
Yes	11	369 (22-996)	2.40 (1.97-2.68)	2.08 (1.64-2.39)

[1] $p < 0.05$ for TBRmax; [2] $p < 0.05$ for TBRmean; [3] [4] $p = 0.075$ for TBRmax and TBR mean. Data shown and median (IQR).
